# It's not just what you say, it's how you say it too. Adolescents' hostile attribution of intent and emotional responses to social comments

**DOI:** 10.1002/ab.21910

**Published:** 2020-06-21

**Authors:** Yvonne H. M. van den Berg, Tessa A. M. Lansu

**Affiliations:** ^1^ Behavioural Science Institute, Social Development Radboud University Nijmegen The Netherlands

**Keywords:** aggression, emotional response, experimental paradigm, hostile attribution bias, youth

## Abstract

A highly prevalent and relevant situation in which adolescents have to interpret the intentions of others is when they interact with peers. We therefore successfully introduced a new paradigm to measure hostile attribution bias (HAB) and emotional responses to such social interactions and examined how it related to youth's aggressiveness. We presented 881 adolescents (*M*_age_ = 14.35 years; *SD* = 1.23; 48.1% male) with audio fragments of age‐mates expressing social comments that varied in content (e.g., what the person says) and tone of voice (e.g., *how* the person says it). Participants' peers also reported on their aggressiveness. In general, added negativity of content and tone was driving the youth's intent attribution and emotional responses to the comments. In line with the Social Information Processing model, we found more hostile attribution of intent and more negative emotional responses of aggressive youth to ambiguous stimuli. Aggression was also related to more hostile intent attributions when both content and tone were negative. Unlike most studies on HAB, the aggression effects in the current study emerged for girls, but not boys. Implications of these results and future use of the experimental paradigm are discussed.

## 1 INTRODUCTION

The Social Information Processing (SIP) model (Crick & Dodge, 1994) has proven to be very useful in understanding youth's aggressive behavior. It explains people's responses to social situations as a function of five steps. The first step for individuals is to encode different social cues. Next, they have to interpret these social cues. Third, people formulate goals and evaluate several responses to the situation to reach these goals. Finally, they select a response and act according to it. Skillful processing at each step is hypothesized to lead to competent performance within a situation, whereas biased processing is likely to result in deviant social behavior (Dodge, 1980). Indeed, research has consistently shown that aggressive children perceive, interpret and make decisions about social stimuli in ways that increase the likelihood of engaging in aggression (see Dodge & Crick, 1990, for a review).

The majority of this work has focused on Step 2 of the SIP model; the interpretation of cues (De Castro, Veerman, Koops, Bosch & Monshouwer, 2002). In a typical study, children are presented with vignette stories describing a variety of social situations in which someone provokes them or causes them a problem. They are then asked about the intentions of the other person. Sometimes, the intent of the other person is clearly negative. Yet in many cases, the intent is more unclear and ambiguous. As a result, children vary in the degree to which they interpret the person's intentions as benign or hostile. A large body of research demonstrates that aggressive children and adolescents tend to attribute more hostile intentions to others, especially when responding to ambiguous social situations (e.g., De Castro, Veerman, Koops, Bosch & Monshouwer, 2002; Verhoef, Alsem, Verhulp, & de Castro, 2019). This phenomenon is referred to as a hostile attribution bias (HAB). The goal of the current study is to introduce a measure for HAB that more closely resembles youth's everyday experiences with ambiguous social situations.

## 2 MEASURING HAB IN EVERYDAY SITUATIONS

When using vignettes (either presented in written text, read aloud by the experimenter, or played from audiotape), children have to imagine that they are in an ambiguous situation. They are not directly experiencing the situation themselves, they have time to process all information, and have time to come up with an informed choice on how to respond. In real life, however, children find themselves suddenly in an ambiguous situation. They have limited time and opportunity to fully process the situation and weight available information before coming to an interpretation and ultimately response. More directly experiencing the situation or being personally involved has therefore been proposed to be an important factor in the manifestation of the HAB (De Castro, Veerman, Koops, Bosch & Monshouwer, 2002; Verhoef et al., 2019). However, only a few studies have used a paradigm in which participants are personally involved or even experience such situations in real‐time (Dodge, 1980; van Dijk, Thomaes, Poorthuis, & de Castro, 2019; Yaros, Lochman, Rosenbaum, & Jimenex‐Camargo, 2014). Given these limitations of traditional vignette measures, researchers also encourage the development of alternative paradigms or techniques to measure hostile intent attributions (De Castro, 2004; Fontaine, 2007; Mize & Pettit, 2008).

In addition, although situations described or enacted in previous measures of HAB are relevant, they are not that highly prevalent in youth's everyday lives. For instance, not being invited to a party (as described in many vignettes), maybe a relevant but not a very prevalent ambiguous social experience. A highly prevalent and relevant situation in which adolescents have to interpret the intentions of others is when they interact with peers and are confronted with comments their peers make. They for example may make comments about the way you look or how you act. These comments can be clearly positive or negative, but are often more ambiguous and require youth to interpret the available information. As youth often encounter comments of peers, such situations likely contribute to the formation of cognitive representations, forming their SIP database. In turn, this has the potential to greatly influence the individual's future social functioning. If someone has the tendency to interpret social information in a more hostile manner (HAB), it is likely that they will also interpret comments made by peers in a hostile manner. As a result, they may respond in retaliatory and aggressive ways toward peers. This will instigate more negative social interactions, which youth then can interpret as confirmation of the earlier hostile interpretation (e.g., that peer made fun of me or tried to be mean). All in all, HAB in everyday interactions may quickly result in a negative cycle of hostile interpretations and responses towards peers. We, therefore, developed a measure in which youth listen to audio fragments of age‐mates who directly address them with an everyday social comment. As such, youth experience the situation rather than having to imagine that they are in a certain situation.

The comments presented to participants not only vary in content (e.g., *what* the person says), but also in the tone of voice (e.g., *how* the person says it). Both the content and tone of voice could add to the communicated intentions of the speaker and may thus affect adolescents' interpretation. In particular, certain combinations of content and tone of voice may be more easily perceived as hostile by youth in general, or by aggressive youth in particular. There are two ways in which content‐tone combinations are most likely to lead to negative interpretations. The first way is through added negativity. When negative content is communicated in a negative tone of voice, the communicated intentions of the speaker are clearly negative. This type of stimulus may, therefore, evoke the strongest negative interpretations among youth. The second way is through ambiguity. One could think of an ambiguous instance where positive content is pronounced in a negative way, which may be interpreted as sarcasm rather than as a sincere compliment. Another ambiguous combination that may be difficult to interpret is when the content is ambiguous and the tone of voice is neutral. In such a situation, there is very little information to base your interpretation on. One's expectations and beliefs about others may be more likely to guide one's interpretation in such situations, possibly leading aggressive youth to make more hostile interpretations.

### 2.1 The role of emotional responses in everyday interactions

In their reformulation of a SIP model for child adjustment, Crick and Dodge (1994) argued that emotion and emotional sensitivity are integral parts of each SIP step. Emotional arousal is a cue that needs to be encoded (Step 1), it influences the interpretation of information (Step 2), influences goal clarification (Step 3), and it can enhance the accessibility of behavioral responses (Step 4 and 5). Moreover, social‐cognitive theories argue that for many children the actual processes leading up to aggression only occur when they are emotionally involved (Lemerise & Arsenio, 2000).

Research has indeed shown that emotional distress leads to more negative interpretations of social cues and subsequent behavioral responses (Lemerise & Arsenio, 2000). Moreover, studies have demonstrated that different emotional responses to ambiguous social situations are associated with different behavioral responses (Crick, 1995; Crick, Grotpeter, & Bigbee, 2002, Mathieson et al., 2011). For example, one study showed that high levels of anger in response to vignettes describing ambiguous social situations is associated with reactive aggression in detained girls (Marsee & Frick, 2007). Moreover, a recent study by Chen, McElwain and Lansford (2019) showed that a HAB in combination with high emotional intensity predicts more negative interactions with friends, whereas a benign attribution bias in combination with high emotional intensity predicts more positive interactions with friends. In a recent meta‐analysis, they also found that hostile intent attribution is more strongly related to aggression when the social situation elicited higher levels of emotional involvement (Verhoef et al., 2019). To better understand and predict youth's responses in social situations, it is therefore important to take into account emotional processes within the SIP model (Lemerise & Arsenio, 2000). That is why we will measure youth's interpretation as well as emotional responses to potentially negative social comments.

### 2.2 Current study

In the current study, we developed a new paradigm to measure intent attribution and emotional responses to everyday comments. We presented adolescents with audio fragments of age‐mates expressing social comments. These comments varied in content (e.g., *what* the person says; positive, ambiguous, negative) and tone of voice (e.g., *how* the person says it; positive, neutral, negative). Our first questions regarded the effect of content and tone of voice on intent attribution and emotional response. Does the content of the comments affect youth's hostile attributions and emotional responses to the comments? And does tone of voice within a type of content matter for the intent attributions and emotional responses to the comments? We expected greater hostile intent attributions and more negative emotional responses when the content would contain more negativity. On top of the effect of content, we expected tone of voice to also affect intent attribution and emotional response in an “added negativity” fashion.

Our second set of questions pertained to how intent attributions and emotional responses to the different content‐tone of voice combinations related to youth's aggression. Is there a hostile interpretation bias or negative emotional response tendency in aggressive youth for certain content‐tone combinations? Based on vignette research, we expect the differentiation between youth high and low in aggression with regard to intent attribution and emotional response to becoming most visible in highly ambiguous situations. This would mean that aggressive youth are expected to respond with more hostile intent attribution and negative emotionality than youth low in aggression for statements that are positive in content, but non‐positive in a tone of voice, and for statements that are ambiguous in content but expressed in a neutral tone of voice.

Finally, we explored whether these associations are moderated by gender. The ambiguous comments in the current study may show greater resemblance to relational aggression scenarios than physical aggression scenarios, as the hurting caused by the other's actions in both the spoken sentences and the relational aggression vignettes seems to be emotional, rather than physical. We therefore mainly base or expectations regarding the role of gender in the association between aggression and the intent attribution and emotional responses on previous work concerning hostile attributions in relationally aggressive situations. Relational aggression has been described to be more normative than physical aggression for girls (Crick & Grotpeter, 1995; Underwood, 2003) and girls show more negative emotional responses to relationally aggressive scenarios than boys (Crick, 1995). Moreover, own relational aggression tends to be associated with hostile attributions in relational aggression scenarios (Crick, 1995; Crick et al., 2002). However, Martinelli, Ackermann, Bernhard, Freitag and Schwenck (2018) do not find strong evidence for relational aggression and a HAB in relational aggression situations to be more strongly associated with girls than boys, when reviewing the current literature. Our examination of moderation by gender in the association between aggression and hostile interpretations and emotional responses, therefore, is exploratory.

We examine these processes in adolescents, as social and cognitive changes occur during this developmental time that increases youth's self‐consciousness (Alfano, Beidel, & Turner, 2002; Westenberg, Siebelink, & Treffers, 2001). Adolescents become more concerned with how one “comes across” in social interactions (Ollendick & Hirshfeld‐Becker, 2002), and fear of negative evaluation seems to increase (Weems & Costa, 2005; Westenberg, Gullone, Bokhorst, Heyne, & King, 2007). This heightened sensitivity to how others evaluate you may make adolescents even more prone than other age groups to ascribe meaning and intentions to the things other people say to them.

### 2.3 Pilot study

A pilot study was conducted to develop a database with auditory stimuli of positive, negative, and ambiguous everyday comments to be used in the main study.

### 2.4 Step 1: Preliminary list of comments

The authors and a research assistant made a preliminary list of comments that people commonly make towards another person in everyday social encounters. They first made their own list of comments, which they then merged into one list consisting of 57 common everyday comments. The content of each comment could be clearly positive (e.g., “nice new shoes”), clearly negative (e.g., “what an ugly sweater”), or potentially ambiguous (e.g., “you are so special”).

### 2.5 Step 2: Selection of comments

A written list of everyday comments was presented to 59 young adults (28.8% male). They were recruited on campus and received course credits for participation. They were on average 20.17 years old (*SD* = 2.55) and 96.6% of the participants were born in the Netherlands. Participants had to be proficient in Dutch.

The respondents had to indicate whether the content of the comment was ambiguous and could be interpreted in more than one way (0 = *no*, 1 =  *yes*). Moreover, they were asked whether they thought the intent of the comment was positive, neutral, or negative using a 7‐point scale (−3 = *very negative,* 0 = *neutral,* +3 = *very positive*). The three comments that a) were least often mentioned as ambiguous and (b) were on average rated as most positive with the smallest standard deviation, were selected as positive comments. The three comments that (a) were least often mentioned as ambiguous and (b) were on average rated as most negative with the smallest standard deviation, were selected as negative comments. The three comments that (a) were most often mentioned as ambiguous and (b) were on average rated as neutral with the largest standard deviation, were selected as ambiguous comments (see Supporting Information Appendix SI).

### 2.6 Step 3: Audio recordings of comments

Finally, audio recordings of the nine comments were made. Each comment was being recorded when it was said out loud by one boy and one girl (both 14 years of age) from a theater school. They were asked to pronounce each comment in a positive, a negative, and a neutral tone of voice. This resulted in a database of 27 auditory stimuli representing all possible combinations of the comments' content (e.g., positive, negative, ambiguous) and tone of voice (e.g., positive, negative, neutral; seeSupporting Information Appendix SI).

## 3 MAIN STUDY

### 3.1 Method

#### 3.1.1 Participants

Participants took part in the 5th wave of the Kandinsky Longitudinal Study, a longitudinal study on detecting children at risk for social and emotional problems in secondary education (van den Berg, Burk, & Cillessen, 2019). The original sample included 1049 adolescents in a large secondary school in the south‐eastern Netherlands. Data of 168 adolescents (16%) was missing due to time constraints (*n* = 142) or technical issues (*n* = 26). As a result, complete data were available for 881 participants (84% of the original sample). Adolescents with incomplete data did not significantly differ from the final sample on the demographic characteristics or any of the main study variables.

Adolescents' mean age was 14.35 years (*SD* = 1.23, range 11.65‐17.71 years) and 48.1% was male. They were in 41 7th to 10th grade classrooms (*M*_classroom size_ = 26.49, *SD* = 2.75, range 18‐30 students), which are the 1st to 4th year of secondary education in the Netherlands. The majority of the adolescents were born in the Netherlands (96.6%) or had parents who were both born in the Netherlands (78.9%).

### 3.2 Measures

#### 3.2.1 Attribution of intent and emotional response

The 27 stimuli were divided into three sets of nine stimuli. Each set consisted of nine different comments (e.g., three positive comments, three negative comments, and three ambiguous comments). Yet, the tone of voice of each comment varied in each set. For instance, the comment “Nice new shoes” (i.e., positive content) was said in a positive tone in set A, said in a negative tone in set B, and in a neutral tone in set C (see Supporting Information Appendix SI). Within each set, every possible combination of content and tone of voice occurred once. Adolescents listened to one set of spoken comments. By doing this, participants would hear all possible combinations of content and tone of voice without being presented with the same comment more than once. Stimuli were presented in random order.

Adolescents were asked to listen to the auditory stimuli and imagine that the comments were directed towards them. They listened to the audio recordings of the same‐sex actor (e.g., boys listened to the voice of the boy actor, girls listened to the girl actor). After hearing each comment, adolescents were asked about the intent of the person making the comment by asking them “When someone says this to me, he probably means this in a … way” (1 = *mean*, 7 = *friendly*). A lower rating indicated a more hostile attribution of intent. After hearing each comment, adolescents were also asked about their emotional response by asking them “When someone says this to me, I find this.” (1 = *unpleasant*, 7 = *pleasant*). A lower rating indicated a more negative emotional response

#### 3.2.2 Aggression

Computerized peer nominations were used to assess adolescents' level of aggression. Each nomination question was presented on a separate screen at the top of the page, followed by a roster with the names of all classmates. Adolescents could nominate classmates by clicking on their names. The order of names was randomized for each participant but kept constant across the questions. Participants could name as many or as few classmates as they wanted, with a minimum of one. Same‐sex and other‐sex nominations were allowed. They could not nominate themselves, as their names were not presented on the screen (for psychometric properties, see van den Berg & Cillessen, 2013).

To measure aggression, seven questions were used, namely “Who kick, hit, or push others?”, “Who call others names?”, “Who say bad things or gossip about others?”, “Who neglect or exclude others?”, “Who feel threatened or attacked easily, even though this might not have been intended? These classmates are not able to control their behavior and feelings and react with aggressive behavior, like yelling or hitting”, “Who try to reach their goals by using aggressive behavior? These classmates intimidate, manipulate or bully others to get admiration, respect or objects”, and “Who bully others?”. The number of nominations received for each question was counted and standardized within classrooms (Cillessen & Marks, 2011). Next, the standardized scores across the seven questions were averaged to one composite score for aggression (Cronbach's *α* = .92).

### 3.3 Procedure

The Kandinsky Longitudinal Study started in 2010 as a close collaboration between teachers of a school and a group of researchers (see (van den Berg, Burk, & Cillessen, 2019)). The school asked whether the researchers could voluntarily help them to assess students' social‐emotion wellbeing. In return, anonymized data were made available to the researchers for scientific purposes.

Each year, the head of the school formally requested the assessment and claimed responsibility for the parental consent procedure. The school requested parental permission at the beginning of the school year for all assessments that they considered necessary for the well‐being of the students. The school distributed a letter among the parents in which the purpose and procedures of the longitudinal research project were described. The letter also requested them to respond if they wanted to exclude their child from participation (e.g., passive informed consent). None of the parents objected to the participation of their child. Adolescents were asked to give assent at the start of the assessment (e.g., active informed assent). None of the participants declined to fill out the questionnaire before or during the assessment. Procedures were approved by the Institutional Review Board of the (van den Berg, Burk, & Cillessen, 2019) (ECG2012‐2505‐038).

Adolescents completed all measures on individual 10‐inch netbook computers during a 45–60‐min classroom session. Participants wore headphones when completing the auditory task. Before assessment, one of the researchers explained the goal and set up of the study. Moreover, participants were explained that the data would be processed anonymously and handled confidentially. Participants were therefore asked to keep their answers to themselves and to be truthful in answering all questions. They were not allowed to talk to other classmates during the assessment, but they could ask questions to one of the researchers and stop participating at any time during the assessment.

## 4 RESULTS

### 4.1 Intent attribution: Valence of content

To examine whether intent attribution varied by the content of the comment, repeated measures analysis of variance (ANOVAs) with a valence of the content of the comments (positive, ambiguous, negative) as the within‐subjects factor were conducted for intent attribution (see Table 1). Content valence mattered, *F*(1.73, 1520.56) = 1192.07; *p* < .001; $\eta_{p}^{2}$ = 0.58. Post‐hoc analyses showed that all means differed significantly with negative comments being perceived as the most hostile, followed by ambiguous comments, followed by positive comments.

**Table 1:** Means and standard deviations for all content‐tone of voice combinations, across the whole sample and per gender

		Positive		Positive		Positive		Ambiguous		Ambiguous		Ambiguous		Negative		Negative		Negative	
Content		Positive		Neutral		Negative		Positive		Neutral		Negative		Positive		Neutral		Negative	
Tone of voice		*M*	*SD*	*M*	*SD*	*M*	*SD*	*M*	*SD*	*M*	*SD*	*M*	*SD*	*M*	*SD*	*M*	*SD*	*M*	*SD*
**Intent attribution**	Boys	5.33	1.64	5.31	1.64	4.17	1.88	3.94	1.75	3.69	1.71	3.16	1.60	2.82	1.70	3.10	1.92	2.86	1.70
	Girls	5.73	1.27	4.93	1.64	4.07	1.92	3.92	1.82	4.05	1.72	3.07	1.64	2.90	1.77	2.85	1.61	2.35	1.38
	Overall	5.54	1.47	5.11	1.65	4.12	1.90	3.93_a_	1.78	3.88_a_	1.72	3.11	1.62	2.86_b_	1.74	2.97_b_	1.77	2.59	1.56
**Emotional response**	Boys	5.04	1.31	5.07	1.37	4.38	1.47	4.11	1.40	3.96	1.40	3.53	1.33	3.23	1.36	3.34	1.35	3.25	1.29
	Girls	5.47	1.16	4.86	1.30	4.24	1.51	3.91	1.67	4.08	1.50	3.24	1.44	2.88	1.36	2.98	1.26	2.67	1.26
	Overall	5.26	1.25	4.96	1.34	4.31	1.49	4.01_a_	1.55	4.02_a_	1.45	3.38	1.40	3.05_b,c_	1.37	3.15_b_	1.32	2.95_c_	1.31

*Note:* *n* boys = 424, *n* girls = 457. Means that differed significantly between boys and girls are underlined.

Overallmeans *within content valence* that did *not* differ significantly in the post hoc *t* tests are marked with identical subscripts.

### 4.2 Intent attribution: Tone of voice within content

To test whether the tone of voice of each comment mattered for the intent attribution given the valence of the content, another set of repeated measures ANOVAs was conducted for each comment valence type with the tone of voice (positive, neutral, negative) as a within‐subjects factor (see Table 1). The tone of voice mattered for intent attribution when presented with positive content comments, *F*(1.95, 1717.31) = 209.67; *p* < .001, $\eta_{p}^{2}$ = 0.19. Positive comments expressed in a negative tone of voice were perceived as most hostile, followed by a neutral tone of voice, followed by a positive tone of voice. The tone of voice also mattered for attribution of intent when presented with ambiguous content comments, *F*(1.94, 1709.58) = 63.41, *p* < .001, $\eta_{p}^{2}$ = 0.07. For ambiguous content, a negative tone of voice was perceived as more hostile than the neutral and positive tone of voice, but the neutral and positive tone of voice were perceived as equally hostile. Finally, tone of voice mattered for attribution of intent when presented with negative comments, *F*(1.97, 1730.13) = 11.68, *p* < .001, $\eta_{p}^{2}$ = 0.01. For negative content, a negative tone of voice was perceived as more hostile than the neutral and positive tone of voice, but the neutral and positive tone of voice were perceived as equally hostile.

### 4.3 Intent attribution: Aggression and gender

Next, we examined whether intent attribution varied by adolescents' gender and level of aggression. For each of the nine content‐tone of voice combinations, a regression was run on intent attribution with gender (boy = 0, girl = 1), aggression, and the interaction between both as predictors (see Table 2). With regard to effects of gender, boys made more hostile attributions than girls when presented with positive comments expressed in a positive tone of voice and when presented with ambiguous comments expressed in a neutral tone of voice. Girls made more hostile attributions than boys when presented with positive comments expressed in a neutral tone of voice and when presented with negative comments expressed in a neutral or negative tone of voice. Aggression was associated with the attribution of intent for ambiguous comments expressed in a neutral tone of voice; more aggressive youth made more hostile attributions.

**Table 2:** Results from hierarchical linear regression analyses predicting attribution of intent from gender and aggression

	Positive						Ambiguous						Negative					
Content	Positive		Neutral		Negative		Positive		Neutral		Negative		Positive		Neutral		Negative	
Tone of voice	*β*	*R* ^2^	*β*	*R* ^2^	*β*	*R* ^2^	*β*	*R* ^2^	*β*	*R* ^2^	*β*	*R* ^2^	*β*	*R* ^2^	*β*	*R* ^2^	*β*	*R* ^2^
Step 1		.02*		.01*		.00		.00		.02*		.00		.00		.00		.03*
Gender	.13*		−0.11*		−.02		.00		.08*		−.02		.03		−.08*		−.16*	
Aggression	−.03		.01		.03		.01		−.08*		.03		.02		−.04		.00	
Step 2		.00		.00		.01*		.00		.01*		.00		.00		.00		.01*
Gender × Aggression	−.03		.03		−.12*		.03		−.11*		−.02		−.08		.03		−.09*	
Total		.02*		.01*		.01*		.00		.03*		.00		.01		.01		.03*

*Note:* *N* = 881.

* *p* < .05.

**Figure 1:**
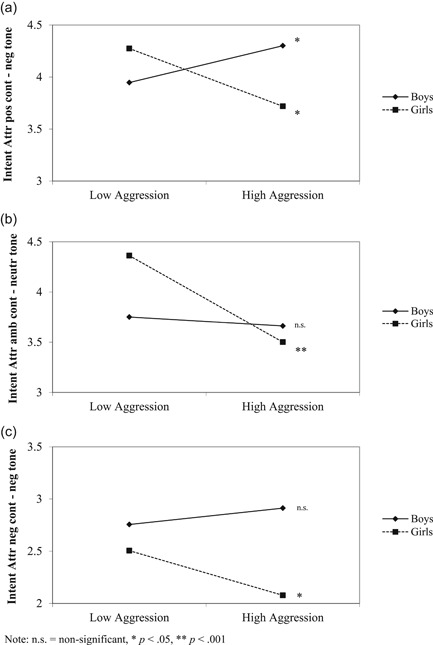
(a) Interaction effect of gender by aggression on intent attributions for positive comments expressed in a negative tone of voice. (b) Interaction effect of gender by aggression on intent attributions for ambiguous comments expressed in a neutral tone of voice. (c) Interaction effect of gender by aggression on intent attributions for negative comments expressed in a negative tone of voice. Note: n.s. =  nonsignificant, * *p* < .05, ** *p* < .001

In addition, interaction effects of gender by aggression were found for various content‐tone combinations. The relation between aggression and attribution of intent differed between boys and girls for positive comments expressed in a negative tone of voice, for ambiguous comments expressed in a neutral tone of voice, and for negative comments expressed in a negative tone of voice. For positive content that was expressed in a negative tone of voice, aggression predicted hostile intent attribution for girls (*r* = ‐.10, *p* = .037), but friendly intent attribution for boys (*r* = .11; *p* = .21; Figure 1a). For ambiguous content that was expressed in a neutral tone of voice, aggression predicted hostile intent attribution for girls (*r* = −.17; *p* < .001), but was not predictive of intent attribution for boys (*r* = .03; *p* = .53; Figure 1b). For negative content that was expressed in a negative tone of voice, aggression again predicted hostile intent attribution for girls (*r* = ‐.11; *p* = 0.025; Figure 1c), but was not predictive of the intent attribution for boys (*r* = .06; *p* = .26). For all these three types of stimuli, aggression thus seemed to be associated with more hostile attributions in girls, but not boys.

### 4.4 Emotional response: Valence of content

Repeated measures ANOVAs with a valence of the content of the comments (positive, ambiguous, negative) as the within‐subjects factor were conducted for emotional responses (see Table 1). Here content valence mattered as well, *F*(1.65, 1455.83) = 1107.38; *p* < .001; $\eta_{p}^{2}$ = 0.56. Post‐hoc analyses showed that all means differed significantly with negative comments making adolescents feel the worst, followed by ambiguous comments, followed by positive comments.

### 4.5 Emotional response: Tone of voice within content

To test whether the tone of voice mattered for the emotional response to comments within the same valence content, repeated measures ANOVAs were conducted for each comment valence type with the tone of voice (positive, neutral, negative) as a within‐subjects factor (see Table 1).

For emotional response, tone of voice mattered when presented with positive content, *F*(1.97, 1730.99) = 146.07; *p* < .001; $\eta_{p}^{2}\;$ = 0.14. For positive content, all means differed significantly, with a negative tone of voice making adolescents feel the worst, followed by a neutral tone of voice, followed by a positive tone of voice. Tone of voice also mattered for emotional response when presented with ambiguous content, *F*(1.97, 1735.67) = 54.21; *p* < .001; $\eta_{p}^{2}$ = 0.06. For ambiguous content, a negative tone of voice made adolescents feel worse than the neutral and positive tone of voice, but the neutral and positive tone of voice led to similar emotional responses. The tone of voice also mattered for an emotional response when presented with negative content, *F*(1.97, 1729.62) = 6.97; *p* = .001; $\eta_{p}^{2}$ = 0.01. For negative content, a negative tone of voice made adolescents feel worse than a neutral tone of voice, but the neutral and positive tone of voice led to a similar emotional response.

### 4.6 Emotional response: Aggression and gender

Finally, we examined whether emotional response varied by adolescents' gender and level of aggression. For each of the nine content‐tone of voice combinations, a regression was run on emotional response with gender (boy = 0, girl = 1) and aggression as predictors (see Table 3). With regard to the effects of gender, boys had a more negative emotional response than girls to the positive content expressed in a positive tone of voice. Girls had a more negative emotional response than boys to positive content expressed in a neutral tone of voice, ambiguous content expressed in a negative tone of voice, and negative content expressed in all types of the tone of voice.

**Table 3:** Results from hierarchical linear regression analyses predicting emotional response from gender and aggression

	Positive						Ambiguous						Negative					
Content	Positive		Neutral		Negative		Positive		Neutral		Negative		Positive		Neutral		Negative	
Tone of voice	*β*	*R* ^2^	*β*	*R* ^2^	*β*	*R* ^2^	*β*	*R* ^2^	*β*	*R* ^2^	*β*	*R* ^2^	*β*	*R* ^2^	*β*	*R* ^2^	*β*	*R* ^2^
Step 1		.03*		.01*		.01		.01		.00		.01*		.02*		.02*		.05*
Gender	.17*		−.07*		−.03		−.05		.03		−.09*		−.12*		−.13*		−.22*	
Aggression	−.01		.03		.06		.05		−.03		.05		.05		.04		.03	
Step 2		.00		.00		.01*		.00		.01*		.00		.00		.00		.00
Gender × aggression	−.07		.01		−.14*		.07		−.12*		−.01		−.03		.02		−.01	
Total		.02*		.01*		.02*		.01		.01*		.01*		.02*		.02*		.05*

*Note:* *N* = 881.

* *p* < .05.

Aggression was associated with the emotional response to ambiguous comments expressed in a neutral tone of voice; youth who scored higher on aggression had a more negative emotional response. The association between aggression and emotional response differed between boys and girls for positive comments expressed in a negative tone of voice, and for ambiguous comments expressed in a neutral tone of voice. For positive content expressed in a negative tone of voice, aggression predicted more negative emotional responses for girls (*r* = −0.10; *p* = .036), but more positive emotional responses for boys (*r* = .16; *p* = .001). For ambiguous content expressed in a neutral tone of voice, aggression predicted more negative emotional responses for girls (*r* = −.14; *p* = .003), but was not predictive of the emotional response for boys (*r* = .04; *p* = .370).

### 4.7 Aggression subtypes

Despite the high reliability of our composite aggression measure (*α* = .92), one could argue that it is made up of theoretically different forms of aggression, that each could have unique associations with intent attributions and emotional responses. To check whether the aggression effects may be driven by a specific subtype of aggression, all regressions were also run for each subtype (see the tables in Supporting InformationAppendix SII for the results). Overall, the results show that the effects of the composite aggression measure on intent attribution and emotional response are not driven by a subset of aggression indicators, nor that one of the aggression indicators is completely unrelated to the outcome variables.

Additional insights provided by these analyses regard gender differences in the role of relational aggression in intent attributions as well as bullies' emotional responses. Relational aggression may be related to making more hostile attributions to ambiguous content pronounced in a neutral way for boys and girls equally. However, for negative content pronounced in a positive tone of voice, results indicate that relational aggression may actually be driving the effect of aggression being more strongly related to hostile intent attribution for girls than boys. In addition, youth high in bullying seem to be less bothered by positive content pronounced in a negative way, ambiguous content pronounced in a negative way, and negative content pronounced in a positive way than youth low in bullying.

## 5 DISCUSSION

The goal of this study was twofold. First, we wanted to introduce a new paradigm to measure HAB and emotional response that more closely resembles the youth's everyday experiences with ambiguous social situations. The pilot study resulted in a set of social comments that varied in content (e.g. *what* the person says) as well as tone of voice (e.g., *how* the person says it). These stimuli were presented to a large group of adolescents in the main study. Results showed that adolescents' attribution of intent and emotional responses depended on the content as well as tone of voice. In general, added negativity of both content and tone was driving youth's responses to the comments.

To further validate this new measure, our second goal was to examine how intent attributions and emotional responses to the social comments were related to youth aggression and whether the association was different for boys and girls. In line with the SIP model, we found more hostile attribution of intent in aggressive youth when they were presented with ambiguous stimuli. Unlike many other studies on HAB (De Castro, Veerman, Koops, Bosch & Monshouwer, 2002), we found aggression to be related to hostile intent attributions and emotional responses in girls, but not boys.

### 5.1 HAB and emotional responses in everyday interactions

In daily life, adolescents spend a significant amount of their waking hours in the presence of others. As such, they are exposed to or actively involved in numerous social interactions, during which they constantly have to interpret the intentions of others' words. Given the frequent and repetitive exposure, an overly negative intent attribution or emotional response can quickly turn into a negative cycle of social interactions. However, up to now, a research paradigm that exposes participants to these frequent social comments to measure their intent attributions and emotional responses did not exist. This study successfully introduced such a method by exposing participants to various social comments that varied in *what* was said as well as *how* it was said.

The youth responded with increasing negativity when positive comments were expressed in a more negative tone of voice. Yet, when it concerned ambiguous or negative content, they did not distinguish between a neutral or positive tone of voice but did respond more negatively to a negative tone of voice. This confirmed our expectation that content and tone of voice would affect intent attribution and emotional response in an “added negativity” fashion showing that it is not just what you say, it is how you say it too.

Notably, the overall results for the attribution of intent and emotional response were mostly similar. Although conceptually different (Crick & Dodge, 1994), the cognitive appraisal of the speaker's intentions as well as the individual's subsequent emotional response seems to be strongly intertwined based on the current results. It is logical that hostile interpretations will facilitate a more negative emotional response. However, hostile interpretations might not be the only factor determining it. Past experiences with being victimized for example affect emotional response to but not the interpretation of ambiguous situations (Lansu, van Noorden, & Deutz, 2017). Personality factors such as resilience or neuroticism might play a role as well in how individuals differ in their intent attributions and emotional response. Future research should further explore the factors that uniquely contribute to cognitive versus emotional processes within the SIP cycle.

### 5.2 Aggression, HAB, and emotional responses

As numerous studies showed that aggressive youth tend to attribute more hostile intentions to others, our second goal was to further validate the new measure by examining how youth's aggression was related to their intent attributions and emotional responses. In line with the multitude of research on the HAB in ambiguous situations (e.g. De Castro, Veerman, Koops, Bosch & Monshouwer, 2002; Verhoef et al., 2019), youth high in aggression responded with more hostile intent attribution to comments that are ambiguous in content and expressed in a neutral tone of voice. Yet, youths' aggression did not predict intent attribution or emotional response for positive comments expressed in a non‐positive tone of voice. When there is very little information (i.e., ambiguous content and no additional information from the tone of voice), youth high in aggression seemed to fill in the blanks by drawing from their SIP database. As aggressive and externalizing youth tend to have negative expectations with regard to the friendliness and trustworthiness of others (Ladd & Troop‐Gordon, 2003), it does not come as a surprise that these youth make more negative intent attributions when there is little information available. In this case, ambiguity through a lack of information might be a more powerful component guiding hostile intent attribution than is ambiguity through conflicting information (i.e., positive comments in a non‐positive tone of voice).

Finally, this new measure seems especially suitable to detect negative SIP among aggressive girls. In general, aggression predicted more hostility and a more negative emotional response to ambiguous stimuli for girls, but not boys. This pattern emerged both for stimuli that were ambiguous in the sense that there was little information on how to interpret the comment (e.g., ambiguous content and tone of voice), as well as for stimuli that contained conflicting information (e.g., positive content pronounced negatively). In addition, for hostile attribution this pattern also emerged when girls were presented with negative content expressed with a negative tone of voice.

Previously, the association between aggression and hostile attributions of intent has been found to be generally stronger in boys (De Castro, Veerman, Koops, Bosch & Monshouwer, 2002). However, this effect seems to be partly due to many traditional measures describing physically hurtful situations, which are more in line with aggression that is more normative for boys than girls. When vignette measures are used that describe ambiguous relationally rather than physically threatening situations, girls show a more negative emotional response, and children who engage in relational aggression themselves tend to make more hostile attributions (Crick, 1995). The stimuli from the current study resemble such relationally threatening situations more closely than the physical harm vignettes, as the hurting caused by the other's actions in both the spoken sentences and the relational aggression vignettes seems to be emotional, rather than physical. In the current study an association between aggression and a negative intent attribution and emotional response to ambiguous stimuli is found for girls but not boys. This pattern nicely fits with the findings of Gentile, Coyne and Walsh (2011) who show an association between relational aggression and HAB in relational aggression vignettes for girls but not boys. However, there are also numerous other studies using relational aggression vignettes, that did not find the effect of aggression on hostile intent attribution to be stronger in girls than boys (Frick et al., 2003; Godleski & Ostrov, 2010; Hoglund & Leadbeater, 2007; Nelson, Mitchell, & Yang, 2008).

### 5.3 Limitations and future research

This study was successful in introducing a measure for HAB and emotional responses that represent youth's everyday experiences with ambiguous social situations. However, there also are several limitations, some of which may be addressed in future research.

Although the results nicely fit with previous findings from the hostile attribution literature, a limitation is that a relatively low amount of variance is explained by the current analyses. A factor that may account for these very modest effect sizes is the age of the sample studied. HAB effects generally tend to become smaller with increasing age (De Castro, Veerman, Koops, Bosch & Monshouwer, 2002). Although effect sizes were small, the impact can be pervasive. Even small differences in negative intent attribution or emotional response can quickly turn into a negative cycle of social interactions due to the frequency of such situations. Especially when adolescents become increasingly occupied with how others view (Ollendick & Hirshfeld‐Becker, 2002), and evaluate them (Weems & Costa, 2005; Westenberg et al., 2007).

Next, it should be noted that the study is concurrent in nature. As such, we are unable to examine the stability and development of intent attributions and emotional responses to everyday social interactions. As already explained in the introduction, everyday social interactions are likely to contribute to individuals' SIP and emotional responses, which in turn affect their future social functioning. It may be that children who have a history of frequent and severe social difficulties (e.g., victimization, rejection, aggression) will respond in a more hostile manner when they are confronted with more ambiguous comments of peers. As a result, their reactions may evoke more negative responses of peers, which strengthens the formation of negative cognitive representations. Future longitudinal designs are therefore recommended as it will enable us to examine the proposed negative cycle of hostile interpretations and responses towards peers and long‐term functioning in the peer group context.

Relatedly, we were not able to assess the psychometric properties (e.g., internal consistency or test‐retest reliability). This was due to the concurrent nature of the study as well as the design in which we presented the set of stimuli to the participants. As explained in the method section, the 27 stimuli were divided into three sets of nine stimuli and participants listened to one of these sets. As a result, participants heard all possible combinations of content and tone of voice, without being presented with the same comment three times (for instance, “You are so cool” pronounced positively, negatively, as well neutral). This also means that they did *not* hear all three positive comments (e.g., “nice new shoes,” “you are so cool,” and “how beautifully worded”) in a positive tone of voice. If this would have been the case, we could have assessed the internal consistency. Future studies should examine the psychometric properties in more detail by presenting all 27 stimuli (e.g., nine comments pronounced in three different ways) to participants.

In addition, the current study presented participants with verbal information without any additional nonverbal cues. Whereas this “clean” design is beneficial from a research perspective, it does not mimic real‐life situations, as nonverbal cues are part of the complex dynamics of social interaction and as such to SIP. Previous work has shown that the facial expression of a perpetrator plays a role in hostile attributions (Horsley, de Castro, & Van der Schoot, 2010), with children making more hostile attributions when the perpetrator is giving the victim a mean look. Nonverbal behavior such as facial expressions likely also influences how people encode and respond to verbal information. Facial expressions may align with what is being said or may contradict with the content or tone of voice, as such influencing intent attributions and emotional responses. It can be an additional source of ambiguity affecting intent attributions or emotional responses, and adding facial expression information when presenting participants with audio stimuli may be an interesting next step for this line of research.

Finally, future research could examine whether it matters for your intent attributions and emotional response *who* is saying these comments. Lemerise and Arsenio (2000) stated that the emotional valence of the relationship between two individuals affects how information is processed and what behavioral response is enacted. For instance, it would be interesting to see whether adolescents make different interpretations when the comment is made by a liked versus disliked peer (Peets, Hodges, Kikas, & Salmivalli, 2007). Comments may be interpreted in a more hostile way when made a by disliked person compared to the same comment made by a friend. Yet if hostile intent is attributed to the comment, the negative emotional response may be more intense when the comment is made by a friend than by a person you already disliked. Another way to examine whether it matters who is making the comment might be to manipulate group membership by emphasizing the regional or ethnic background of the speaker by varying the accent in which comments are made. This enables researchers to test whether youth respond with a more hostile attribution when comments are made by outgroup compared to ingroup members.

## 6 CONCLUSION

We introduced a new audio paradigm to measure intent attribution and emotional responses to everyday comments, showing that both content and tone of voice matter. This paradigm can be used to study differences in intent attributions, as well as in emotional responses. In line with findings obtained with more traditional measures, aggression proved to be related to intent attributions and emotional response. However, in this study aggressive girls rather than boys showed more negative responses. As the paradigm is experience‐based and closely resembles youth's frequent everyday interactions, it has high ecological validity. As such, it provides numerous opportunities to study youth's SIP during common social encounters.

## Supporting information

Supplementary information

Supplementary information
