# Supplementary material for: It's not just what you say, it's how you say it too. Adolescents' hostile attribution of intent and emotional responses to social comments
Source: Aggress Behav. 2020 Jun 21;46(5):425–36. doi: 10.1002/ab.21910 (PMC7496477; doi:10.1002/ab.21910)
Supplement: Supplementary file 1 — Supplementary information [file AB-46-425-s001.docx]

|  | Comment | | Descriptive statistics (Pilot Study) | | | | Subset (Main Study) | | | |
| --- | --- | --- | --- | --- | --- | --- | --- | --- | --- | --- |
|  |  | | Ambiguous item  (% yes) | Attribution of intent  (-3 = ‘very negative’,  3 = ‘very positive’) | | |  | Tone of voice | | |
|  | Dutch (Original) | English (Translation) |  |  |  |  | Content | Positive | Negative | Neutral |
|  |  |  |  | M | SD |  |  |  |  |  |
| 1 | Wat een leuke nieuwe schoenen | Nice new shoes | 10.17 | 2.07 | .89 |  | Positive | A | B | C |
| 2 | Wat ben jij cool | You are so cool | 37.29 | 1.31 | 1.32 |  | Positive | C | A | B |
| 3 | Wat zeg je dat mooi | How beautifully worded | 32.20 | 1.85 | .87 |  | Positive | B | C | A |
| 4 | Wat een lelijke trui | What an ugly sweater | 3.39 | -2.41 | .70 |  | Negative | B | C | A |
| 5 | Wat ben jij stom | You are stupid | 16.95 | -2.29 | .80 |  | Negative | A | B | C |
| 6 | Wat kijk je boos | You look angry | 18.64 | -1.12 | .85 |  | Negative | C | A | B |
| 7 | Wat heb je met je haar gedaan? | What did you do with your hair? | 71.19 | -.71 | 1.10 |  | Ambiguous | C | A | B |
| 8 | Wat ben jij bijzonder | You are so special | 79.66 | 1.07 | 1.34 |  | Ambiguous | B | C | A |
| 9 | Wat doe jij nou? | What are you doing? | 79.66 | -.90 | .71 |  | Ambiguous | A | B | C |

Appendix I. Auditory Stimuli in Dutch (Original) and English (Translation)
