# Supplementary material for: It's not just what you say, it's how you say it too. Adolescents' hostile attribution of intent and emotional responses to social comments
Source: Aggress Behav. 2020 Jun 21;46(5):425–36. doi: 10.1002/ab.21910 (PMC7496477; doi:10.1002/ab.21910)
Supplement: Supplementary file 2 — Supplementary information [file AB-46-425-s002.docx]

Appendix II

Table 1. *Results from Hierarchical Linear Regression Analyses Predicting Attribution of Intent from Gender and Aggression* *Variables Separately.*

| Content |  | | Positive | |  | |  | | Ambiguous | |  | |  | | Negative | |  | |
| --- | --- | --- | --- | --- | --- | --- | --- | --- | --- | --- | --- | --- | --- | --- | --- | --- | --- | --- |
| Tone of Voice | Positive | | Neutral | | Negative | | Positive | | Neutral | | Negative | | Positive | | Neutral | | Negative | |
|  | β | *Total R²* | β | *Total R²* | β | *Total R²* | β | *Total R²* | β | *Total R²* | β | *Total R²* | β | *Total R²* | β | *Total R²* | β | *Total R²* |
| *Step 1* |  |  |  |  |  |  |  |  |  |  |  |  |  |  |  |  |  |  |
| Gender | .13* |  | -.11* |  | -.01 |  | .01 |  | .07† |  | -.02 |  | .04 |  | -.10* |  | -.17* |  |
| Hits & kicks | -.02 | .02 | .02 | .01 | .05 | .00 | .03 | .00 | -.08* | .02 | .03 | .00 | .04 | .00 | -.06 | .01 | -.01 | .03 |
| Calls names | -.05 | .02 | -.02 | .01 | .04 | .00 | .02 | .00 | -.08* | .02 | .06 | .00 | .03 | .00 | -.04 | .01 | .05 | .03 |
| Gossips | -.01 | .02 | .03 | .01 | .00 | .00 | .02 | .00 | -.07* | .02 | .00 | .00 | -.02 | .00 | -.01 | .01 | -.01 | .03 |
| Excludes | -.01 | .02 | .00 | .01 | .01 | .00 | .02 | .00 | -.07* | .01 | .00 | .00 | .03 | .00 | -.02 | .01 | -.01 | .03 |
| Reactive | -.05 | .02 | .00 | .01 | .02 | .00 | -.02 | .00 | -.04 | .01 | .02 | .00 | -.01 | .00 | -.02 | .01 | -.01 | .03 |
| Proactive | .00 | .02 | .02 | .01 | .04 | .00 | .01 | .00 | -.08* | .01 | .04 | .00 | .04 | .00 | -.04 | .01 | .00 | .03 |
| Bullies | -.02 | .02 | .01 | .01 | .04 | .00 | -.01 | .00 | -.09* | .02 | .06† | .00 | .07† | .00 | -.05 | .01 | .02 | .03 |
| *Step 2* |  |  |  |  |  |  |  |  |  |  |  |  |  |  |  |  |  |  |
| G x Hits & kicks | .00 | .02 | .08† | .02 | -.13* | .01 | .04 | .00 | -.15* | .03 | -.01 | .00 | .01 | .00 | .02 | .01 | -.11* | .03 |
| G x Calls names | -.01 | .02 | .05 | .02 | -.09* | .01 | .08† | .00 | -.14* | .03 | .00 | .00 | -.06 | .00 | .02 | .01 | -.09* | .03 |
| G x Gossips | -.08 | .02 | .03 | .02 | -.13* | .01 | -.01 | .00 | -.07 | .02 | -.09† | .00 | -.17* | .01 | .00 | .01 | -.11* | .03 |
| G x Excludes | -.06 | .02 | .03 | .01 | -.12* | .01 | .03 | .00 | -.05 | .02 | -.02 | .00 | -.09* | .01 | .02 | .01 | -.08† | .03 |
| G x Reactive | .02 | .02 | .03 | .01 | -.07† | .01 | .00 | .00 | -.10* | .02 | .03 | .00 | -.01 | .00 | .07† | .01 | -.05 | .03 |
| G x Proactive | -.04 | .02 | .01 | .01 | -.08* | .01 | .00 | .00 | -.09* | .02 | -.02 | .00 | -.03 | .00 | .00 | .01 | -.06 | .03 |
| G x Bullies | -.05 | .02 | -.01 | .01 | -.08† | .01 | .01 | .00 | -.11* | .03 | -.03 | .00 | -.06 | .00 | .03 | .01 | -.07† | .03 |

*Note*. *N* = 881 * *p* < .05, † *p* < .10

Table 2. *Results from Hierarchical Linear Regression Analyses Predicting Emotional Response from Gender and Aggression* *Variables Separately.*

| Content |  | | Positive | |  | |  | | Ambiguous | |  | |  | | | Negative | | |  | |
| --- | --- | --- | --- | --- | --- | --- | --- | --- | --- | --- | --- | --- | --- | --- | --- | --- | --- | --- | --- | --- |
| Tone of Voice | Positive | | Neutral | | Negative | | Positive | | Neutral | | Negative | | Positive | | | Neutral | | | Negative | |
|  | β | *Total R²* | β | *Total R²* | β | *Total R²* | β | *Total R²* | β | *Total R²* | β | *Total R²* | β | *Total R²* | β | | *Total R²* | β | | *Total R²* |
| *Step 1* |  |  |  |  |  |  |  |  |  |  |  |  |  |  |  | |  |  | |  |
| Gender | .17* |  | -.07^†^ |  | -.02 |  | -.05 |  | .03 |  | -.08* |  | -.10* |  | -.12* | |  | -.22* | |  |
| Hits & kicks | -.01 | .03 | .03 | .01 | .07* | .01 | .03 | .01 | -.03 | .00 | .06^†^ | .01 | .06^†^ | .02 | .04 | | .02 | .02 | | .05 |
| Calls names | -.03 | .03 | -.00 | .01 | .07^†^ | .01 | .06 | .01 | -.03 | .00 | .06 | .01 | .04 | .02 | .05 | | .02 | .04 | | .05 |
| Gossips | .00 | .03 | .04 | .01 | .04 | .00 | .07* | .01 | -.02 | .00 | .02 | .01 | .04 | .02 | .05 | | .02 | .06^†^ | | .05 |
| Excludes | -.01 | .03 | .02 | .01 | .03 | .00 | .07* | .01 | -.03 | .00 | .01 | .01 | .05 | .02 | .02 | | .02 | .03 | | .05 |
| Reactive | .01 | .03 | .01 | .01 | .04 | .00 | -.03 | .00 | .01 | .00 | .04 | .01 | -.02 | .02 | .01 | | .02 | -.04 | | .05 |
| Proactive | .00 | .03 | .02 | .01 | .05 | .01 | .03 | .00 | -.04 | .00 | .07* | .02 | .06^†^ | .02 | .02 | | .02 | .01 | | .05 |
| Bullies | -.01 | .03 | .01 | .01 | .09* | .01 | .04 | .01 | -.04 | .00 | .11* | .02 | .11* | .03 | .06^†^ | | .02 | .04 | | .05 |
| *Step 2* |  |  |  |  |  |  |  |  |  |  |  |  |  |  |  | |  |  | |  |
| G x Hits & kicks | -.00 | .03 | .01 | .01 | -.14* | .02 | .05 | .01 | -.11* | .01 | .06 | .02 | .03 | .02 | .04 | | .02 | .04 | | .05 |
| G x Calls names | -.09* | .04 | -.01 | .01 | -.13* | .02 | .09* | .01 | -.16* | .02 | .00 | .01 | -.05 | .02 | -.00 | | .02 | .00 | | .05 |
| G x Gossips | -.10* | .03 | -.00 | .01 | -.17* | .02 | .04 | .01 | -.10^†^ | .01 | -.05 | .01 | -.10^†^ | .02 | .00 | | .02 | -.04 | | .05 |
| G x Excludes | -.09* | .03 | .00 | .01 | -.14* | .02 | .06 | .01 | -.09* | .01 | -.02 | .01 | -.06 | .02 | .01 | | .02 | -.03 | | .05 |
| G x Reactive | -.01 | .03 | .02 | .01 | -.07^†^ | .01 | .03 | .01 | -.12* | .01 | .01 | .01 | -.02 | .02 | .04 | | .02 | -.04 | | .05 |
| G x Proactive | -.07^†^ | .03 | -.01 | .01 | -.10* | .01 | .05 | .01 | -.08* | .01 | -.00 | .02 | .01 | .02 | -.02 | | .02 | -.01 | | .05 |
| G x Bullies | -.10* | .04 | .00 | .01 | -.10* | .02 | .06 | .01 | -.09* | .01 | .03 | .02 | -.01 | .03 | .00 | | .02 | .01 | | .05 |

*Note*. *N* = 881 * *p* < .05, † *p* < .10
